# 

**Published:** 1903-10-10

**Authors:** 


					the hospital. "The standard of highest purity."?uctobku iu??, iyw.
CADBURY'S rf H ^ CADBURY'S
cocoa )% mm cocoa
"A PERFECT FOOD." ~~~W WO DRUGS OR ALK&UE8
ao
taoir West KAN Infirmary I f J
rol. XXXV. [?gggi] jSgg&fr
ENj
? "-NOMOLKamaJYOH tr/CH
[Smbsoriptloi^Terins,] pR|CE THREEpENQEi

				

